# Trends in the Incidence of Hypertensive Disorders of Pregnancy Among the Medicaid Population before and During COVID-19

**DOI:** 10.1089/whr.2024.0045

**Published:** 2024-09-06

**Authors:** Jessica Lin, Heidi Feng, Ronald Horswell, San Chu, Yun Shen, Gang Hu

**Affiliations:** ^1^ Pennington Biomedical Research Center, Baton Rouge, Louisiana, USA.; ^2^ Freeman School of Business, Tulane University, New Orleans, Louisiana, USA.

**Keywords:** HDP, incidence, COVID-19, Medicaid, health disparities

## Abstract

**Importance:**

Hypertensive disorders of pregnancy (HDP) are a group of high blood pressure disorders during pregnancy that are a leading cause of maternal and infant morbidity and mortality. Data on the trend in the incidence of HDP among the Medicaid population during coronavirus disease of 2019 (COVID-19) are lacking.

**Objective:**

To determine the trends in the annual incidence of HDP among pregnant Medicaid-insured women in Louisiana before and during the COVID-19 pandemic (2016–2021).

**Methods:**

A total of 113,776 pregnant women aged 15–50 years were included in this study. For multiparous individuals, only the first pregnancy was used in the analyses. Women with a diagnosis of each type-specific HDP were identified by using the International Classification of Diseases, 10th revision (ICD-10) codes. The annual incidence of HDP was calculated for each race and age subgroup. For each type-specific HDP, the annual age-specific incidence was calculated.

**Results:**

The incidence of HDP increased from 10.5% in 2016 to 17.7% in 2021. The highest race/ethnicity-specific incidence of HDP was seen in African American women (19.2%), then White women (13.1%), followed by other women (10.7%).

**Conclusion and Relevance:**

HDP remains a very prevalent and significant global health issue, especially in African American women and during the COVID-19 pandemic. Severe HDP substantially increases the risk of mortality in offspring and poses long-term issues for both mother and infant. HDP prevention holds particular relevance for the Medicaid population, given the health care disparities and barriers that impact quality of care, leading to an increased risk for HDP.

## Introduction 

Hypertensive disorders of pregnancy (HDP) are a group of high blood pressure disorders during pregnancy that include gestational hypertension, chronic hypertension, preeclampsia, and preeclampsia superimposed on chronic hypertension.^1,2^ Gestational hypertension is persistent *de novo* hypertension that arises at or after 20 weeks’ gestation in the absence of clinical features of preeclampsia. Chronic hypertension is high blood pressure (systolic blood pressure ≥140 mmHg or diastolic blood pressure ≥90 mmHg) predating the pregnancy or recognized before 20 weeks’ gestation.^2^ Preeclampsia is gestational hypertension accompanied by ≥1 of the following new-onset conditions at or after 20 weeks’ gestation: protein in urine and/or swelling in legs, feet, and hands.^2^ Preeclampsia superimposed on chronic hypertension is chronic hypertension with new-onset proteinuria or other signs/symptoms of preeclampsia after 20 weeks’ gestation or chronic proteinuria with new-onset hypertension.^2^

HDP complicates between 5% and 10% of all pregnancies and is a leading cause of maternal and infant morbidity and mortality.^3–5^ Mothers with HDP have an increased risk of renal damage, placental abruption, hepatic injury, pulmonary edema, and HELLP syndrome.^6^ HELLP syndrome is characterized by the presence of hemolysis, elevated liver enzymes, and low platelet counts.^6^ HDP is also associated with adverse pregnancy and birth outcomes, such as preterm birth, low birth weight, small for gestational age, stillbirth, cesarean section, induced labor, admission to neonatal intensive care units, and perinatal mortality for the infant.^7–9^ Offspring of mothers with severe and early onset preeclampsia had more than six times the mortality risk compared with offspring of mothers with no HDP.^10^

Furthermore, mothers with HDP and their children have increased short-term and long-term risks for diseases and cardiometabolic disorders. Mothers with HDP have an increased risk of future cardiometabolic disease, such as hypertension, coronary heart disease, cardiac arrhythmia, myocardial infarction, type 2 diabetes, and stroke.^4,9^ In addition, HDP is associated with a 50% increased risk for cardiovascular disease (CVD) within 5 years after childbirth and a two to three times higher risk of hypertension between 2 and 7 years postpregnancy.^10^ Moreover, mothers with preeclampsia have a higher risk for CVD mortality within the first 10 years after childbirth.^11^

Common risk factors associated with HDP include obesity, African American ethnicity, lower household income, lower educational level, advanced maternal age, rural residential area, lack of antenatal care, family history of hypertension, diabetes mellitus, sedentary life, and tobacco use.^3,12–15^ Many studies have shown that African American women have a higher risk for HDP.^1,9,16^ In addition, African American women with HDP experience higher rates of adverse outcomes during obstetric care^17,18^ and a higher risk of maternal^1,17^ and intrauterine fetal^18^ mortality compared with White women. Several,^19–21^ but not all, studies^22,23^ show that women who tested positive for coronavirus disease of 2019 (COVID-19) during their pregnancy also had a greater risk of HDP.

The Louisiana population confronts many health challenges as a leading state in the prevalence of CVD, diabetes, obesity, physical inactivity, and tobacco use.^24^ Louisiana has a higher rate in each of the major disease categories (heart disease and stroke, obesity, and diabetes) than the average rate of the United States.^24^ In Louisiana, over 1.9 million people receive health care coverage through Medicaid, with many from low socioeconomic status (SES) households.^25^ Medicaid provides medical benefits and services to individuals and families with low income, and those covered by Medicaid generally have poorer health and health outcomes. In addition, women insured by Medicaid experienced more severe elevated blood pressure and higher rates of adverse neonatal outcomes compared with those with private insurance.^26^

Whereas several studies have assessed the trend in the incidence of HDP, only a few studies have assessed the trend in the incidence of HDP among the Medicaid population.^27^ This study aimed to determine the trends in the annual prevalence of HDP among pregnant Medicaid-insured women in Louisiana from 2016 to 2021 and to compare the incidence of HDP before and during the COVID-19 pandemic.

## Methods

The data source used in this study was from the Louisiana Medicaid program, which operates within the Louisiana Department of Health. The Louisiana Medicaid program covers approximately 38,000 pregnant women and newborn annually and links the dataset between the mother and child. The Centers for Medicaid Services (CMS) collects the Medicaid administrative data, which is derived from reimbursement information of payment of bills. This study included Louisiana Medicaid datasets for all pregnancies between January 1, 2016, and December 31, 2021. The study and analysis plan were approved by both Pennington Biomedical Research Center and the Louisiana Department of Health Institutional Review Boards. We did not obtain informed consent from participants involved in our study because we used anonymized data compiled from electronic medical records.

Women aged 15–50 years during childbirth with a diagnosis of HDP were identified by using the International Classification of Diseases, 10th revision (ICD-10) codes according to the guidelines of the International Society for the Study of Hypertension in Pregnancy (ISSHP). For multiparous individuals, only data from their first pregnancy were included in the analyses. Gestational hypertension was identified using the ICD-10 code O13 and specifically selecting women who were diagnosed at or after 20 weeks’ gestation. Chronic hypertension before pregnancy was identified by the ICD-10 code O10.01 and selecting women who had elevated blood pressure predating the pregnancy or recognized before 20 weeks’ gestation. Preeclampsia was identified by the ICD-10 code O14.0 and selecting women who were diagnosed at or after 20 weeks’ gestation. Preeclampsia superimposed on chronic hypertension was identified by the ICD-10 code O11.0 and selecting for women who were diagnosed before 20 weeks’ gestation.

**FIG. 1.**
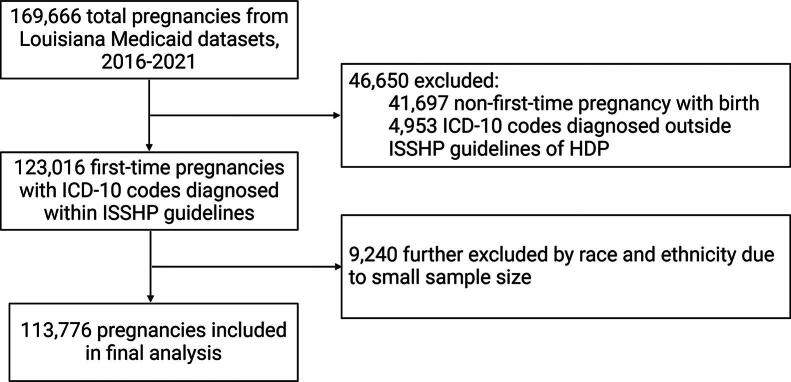
Flow diagram of the study.

For race/ethnicity-specific analyses, the three largest race/ethnicity sample sizes were selected: White, Black, and others. The other races/ethnicities (Asian, Hawaiian or Pacific Islander, and Native American) were excluded due to a smaller sample size (*e.g., n* = 1,685 for Asian women throughout the study period). After excluding records of non-first-time pregnancies, ICD-10 codes of HDP diagnosed outside of the International Society for the Study of Hypertension in Pregnancy guidelines, and race and ethnicities with a small sample size, the final analysis included 113, 776 individuals (Fig. 1).

### Statistical analysis

The number of pregnancies and incidence for HDP overall and each type-specific HDP of each study year and age group were reported. The general linear model was used to calculate the yearly age-specific incidence of HDP and yearly race/ethnicity-specific incidence of HDP. The linear trend in incidence across time was tested using the incidence of total or type-specific HDP as the outcome variable and year as a continuous by using the logistic regression. Direct standardization was used to calculate the age-standardized incidence to the 2010 Census population using the following age groups: 15–19, 20–24, 25–29, 30–34, 35–39, and 40–50 years old. All statistical analyses are performed with SAS Software Version 9.4 (SAS Institute Inc.). The results were considered statistically significant when a two-sided *P* < 0.05.

## Results

The number of first-time pregnancies for each age group and study year from 2016 to 2021 are shown in Table 1. Our study consisted of 113,776 first-time pregnant women. The average maternal age during childbirth was 26.4 ± 6.0 years and was not significantly different before and during the COVID-19 pandemic. The age group of 40–50 years had the smallest sample size, and the age group of 20–24 years had the largest sample size. The average age during childbirth decreased from 26.7 ± 5.64 years old in 2016 to 25.8 ± 6.03 years old in 2021.

**Table 1.** Numbers of First Pregnancy by Age and Year in Louisiana Medicaid Population from 2016 to 2021

	Years of first pregnancy					
Age group (years)	2016	2017	2018	2019^a^	2020^a^	2021
No. of first-time pregnancies						
15–19	2288	2339	2535	3236	2316	2713
20–24	6528	5902	5869	7189	5742	7265
25–29	6359	5438	4731	4918	3458	3710
30–34	3974	3654	3281	3581	2387	2702
35–39	1657	1637	1683	1813	1333	1457
40–50	342	345	359	370	311	354
Total	21,148	19,315	18,458	21,107	15,547	18,201
Age during childbirth, mean (SD)	26.7 (5.64)	26.7 (5.85)	26.5 (6.05)	26.1 (6.06)	26.0 (6.12)	25.8 (6.03)

^a^ The 2019 dataset consisted of dates from 2019 until the COVID-19 pandemic in February 2020. The 2020 dataset consisted of dates from March 2020 until December 2020.

The annual prevalence of HDP for each age group is shown in Table 2. The crude incidence increased from 7.7% in 2016 to 13.2% in 2021, and the age-standardized rate increased from 10.5% in 2016 to 17.7% in 2021 (*P* for trend <0.001). The highest incidence of HDP for each study year was seen in the highest maternal age group, 40–50 years. The incidence of HDP was higher in the years during the COVID-19 pandemic, with only one exception (age group of 35–39 years in 2020).

**Table 2.** Incidence (%) of Hypertensive Disorders of Pregnancy by Age and Year in Louisiana Medicaid Population from 2016 to 2021

Age group (years)	Years of first pregnancy						*p* Values
	2016	2017	2018	2019^a^	2020^a^	2021	
15–19	7.0	7.4	8.7	9.4	10.9	10.3	<0.001
20–24	6.1	8.2	9.1	9.4	10.8	11.3	<0.001
25–29	7.0	8.8	9.7	11.2	12.2	13.5	<0.001
30–34	9.3	11.4	13.0	14.5	15.1	15.4	<0.001
35–39	11.6	14.3	15.8	19.2	16.9	19.7	<0.001
40–50	15.5	17.4	24.2	23.2	25.4	25.7	0.001
Total	7.7	9.5	10.8	11.8	12.6	13.2	<0.001
Age-standardized^b^	10.5	12.4	15.3	16.1	17.0	17.7	<0.001

^a^ The 2019 dataset consisted of dates from 2019 until the COVID-19 pandemic in February 2020. The 2020 dataset consisted of dates from March 2020 until December 2020.

^b^ Age adjusted for the direct method to the year 2000 Census population using the age groups 15–19. 20–24, 25–29, 30–34, 35–39, and 40–50 years.

The annual incidence of each type specific HDP for each age group is shown in Table 3. The crude incidence for gestational hypertension increased from 1.6% in 2016 to 2.3% in 2021, and the age-standardized rate had an overall increase from 2.0% in 2016 to 2.6% in 2021 (*P* for trend <0.001). The crude incidence for chronic hypertension before pregnancy had an overall increase from 1.1% in 2016 to 1.9% in 2021, and the age-standardized rate had an overall increase from 2.1% in 2016 to 3.8% in 2021 (*P* for trend <0.001). The crude incidence for preeclampsia increased from 3.6% in 2016 to 7.1% in 2021, and the age-standardized rate increased from 4.0% in 2016 to 7.9% in 2021. The crude incidence for chronic hypertension with superimposed preeclampsia had an overall increase from 1.3% in 2016 to 1.9% in 2021, and the age-standardized rate had an overall increase from 2.4% in 2016 to 3.8% in 2020 and then decreased to 3.3% in 2021 (*P* for trend <0.001). The prevalence of each type specific HDP had an overall increase as the age group increased. The age-standardized incidence of obesity significantly increased from 18.4% in 2016 to 31.4% in 2021 (*P* for trend <0.001), while the incidence of preexisting diabetes remained stable from 2.8% in 2016 to 2.3% in 2021.

**Table 3.** Incidence (%) of Type-Specific Hypertensive Disorders of Pregnancy by Age and Year in Louisiana Medicaid Population from 2016 to 2021

	Years of first pregnancy						
Age group (years)	2016	2017	2018	2019^a^	2020^a^	2021	*p* Values
Gestational hypertension							
15–19	1.4	1.3	2.2	1.9	1.6	2.3	0.044
20–24	1.4	2.0	2.1	2.2	2.2	2.2	0.004
25–29	1.5	1.6	1.7	2.1	2.5	2.5	<0.001
30–34	2.0	2.0	2.1	1.5	2.5	2.3	0.175
35–39	1.8	1.5	1.6	2.8	1.9	2.3	0.055
40–50	2.9	2.0	2.8	2.4	3.9	3.4	0.767
Total	1.6	1.8	2.0	2.1	2.2	2.3	<0.001
Age-standardized^b^	2.0	1.8	2.2	2.2	2.7	2.6	<0.001
Chronic hypertension before pregnancy							
15–19	0.5	0.3	0.2	0.4	0.5	0.4	0.326
20–24	0.5	0.7	0.7	0.8	0.9	0.9	0.052
25–29	1.0	1.7	1.8	1.8	1.8	2.3	<0.001
30–34	1.6	2.8	3.1	3.6	3.2	3.3	<0.001
35–39	3.1	4.4	4.7	5.4	4.2	4.5	0.045
40–50	3.8	5.2	8.1	5.4	4.5	7.3	0.141
Total	1.1	1.7	1.8	1.9	1.7	1.9	<0.001
Age-standardized^b^	2.1	3.0	4.0	3.4	2.9	3.8	<0.001
Preeclampsia							
15–19	4.8	5.3	6.0	6.3	8.1	7.1	<0.001
20–24	3.5	4.7	5.3	5.6	6.7	7.0	<0.001
25–29	3.3	4.3	4.3	5.4	6.5	6.6	<0.001
30–34	3.6	4.4	4.4	5.9	6.1	7.1	<0.001
35–39	3.9	4.9	5.6	6.2	6.8	7.6	<0.001
40–50	4.4	4.4	6.1	8.1	9.0	9.9	0.011
Total	3.6	4.6	5.0	5.8	6.8	7.1	<0.001
Age-standardized^b^	4.0	4.6	5.4	6.6	7.5	7.9	<0.001
Chronic hypertension with superimposed preeclampsia							
15–19	0.4	0.6	0.4	0.8	0.7	0.6	0.243
20–24	0.8	0.8	1.1	0.8	1.0	1.2	0.026
25–29	1.2	1.2	1.9	2.0	1.4	2.1	<0.001
30–34	2.1	2.2	3.3	3.4	3.4	2.8	<0.001
35–39	2.8	3.5	3.9	4.9	4.0	5.2	0.007
40–50	4.4	5.8	7.2	7.3	7.7	5.1	0.368
Total	1.3	1.5	2.0	2.0	1.8	1.9	<0.001
Age-standardized^b^	2.4	3.0	3.7	2.9	3.8	3.3	<0.001

^a^ The 2019 dataset consisted of dates from 2019 until the COVID-19 pandemic in February 2020. The 2020 dataset consisted of dates from March 2020 until December 2020.

^b^ Age adjusted for the direct method to the year 2000 Census population using the age groups 15–19. 20–24, 25–29, 30–34, 35–39, and 40–50 years.

The annual race/ethnicity-specific incidence of HDP for each age group is shown in Table 4. The race/ethnicity-specific incidence of HDP has increased over the course of the study years, with the highest incidences seen during the COVID-19 pandemic. The mean maternal age during childbirth varied from 27.8 years in other women to 26.2 years in African American women and 25.9 years in White women. The age-standardized rate showed the highest incidence in African American women (19.2%) followed by White women (13.1%) and other women (10.7%) (*P* for trend <0.001). The incidence of HDP showed the greatest increase across the study period in White women (5.9%), then African American women (5.3%) and women of other women (4.5%).

**Table 4.** Annual Race/Ethnicity-Specific Incidence (%) of Hypertensive Disorders of Pregnancy by Age and Year in Louisiana Medicaid Population from 2016 to 2021

	White		African American		Other		
Year pregnancy	No./No.	Incidence	No./No.	Incidence	No./No.	Incidence	*p* Values
2016	628/9752	6.4	833/8176	10.2	117/2295	5.1	<0.001
2017	715/8953	8.0	954/7542	12.7	129/2039	6.3	<0.001
2018	832/8516	9.8	966/7398	13.1	142/1820	7.8	<0.001
2019^a^	899/8729	10.3	1285/8798	14.6	255/3064	8.3	<0.001
2020	684/6108	11.2	1021/6731	15.2	227/2394	9.5	<0.001
2021	910/7378	12.3	1190/7695	15.5	265/2755	9.6	<0.001
Total	4,668/49,436	9.4	6,249/46,340	13.5	1,135/14,367	7.9	<0.001
Age-standardized^b^		13.1		19.2		10.7	<0.001
*p* Values (trend)		<0.001		<0.001		<0.001	

No./No., numbers of GDM cases/numbers of first-time pregnancies.

^a^ The 2019 dataset consisted of dates from 2019 until the COVID-19 pandemic in February 2020. The 2020 dataset consisted of dates from March 2020 until December 2020.

^b^ Age adjusted for the direct method to the year 2000 Census population using the age groups 15–19. 20–24, 25–29, 30–34, 35–39, and 40–50 years.

## Discussion

### Main findings

In this present study, the age-standardized incidence of HDP increased from 10.5% in 2016 to 17.7% in 2021. The trend seen in this study is in congruence with findings from both national and global data.^1,28–30^ In Denmark, offspring of mothers with preeclampsia and eclampsia had an increased risk of all-cause mortality by 29% and 188%, respectively.^28^ In this present study, the annual incidence of HDP increased for each race/ethnicity. The overall incidence of HDP was also higher during the COVID-19 pandemic compared with preceding years.

Before the COVID-19 pandemic, the incidence of HDP among the Louisiana Medicaid population increased, likely due to a rise in HDP risk factors such as obesity, preexisting diabetes, and preexisting chronic hypertension. According to the Louisiana Department of Health Diabetes and Obesity action report, the number of Louisiana Medicaid enrollees aged 21 years and older diagnosed with obesity increased annually from 31,830 individuals to 99,207 individuals from 2016 to 2018.^31,32^ During the same period, the number of Louisiana Medicaid women diagnosed with diabetes increased from 14,090 to 53,849.^31–33^

The findings in our study showed the highest age-standardized rate and crude incidence of HDP during the COVID-19 pandemic. Pregnant women with COVID-19 had an increased risk of adverse pregnancy outcomes, such as preeclampsia, preterm birth, and stillbirth.^33^ COVID-19 led to an inactive lifestyle with lockdown restrictions to limit direct contact between individuals and, therefore, reduce exposure to the virus.^34^ Previous studies have attested to an increased risk of hypertension during COVID-19, with sedentary behavior as one of the principal risk factors for new-onset and worsening of hypertension.^35,36^ A national survey reported that home confinement during the pandemic led the daily sitting time to increase from 5 to 8 hours (28.6%).^37^ Physical activity is also associated with a significantly reduced risk of gestational hypertensive disorders.^38^ During the COVID-19 pandemic, fewer young adults aged 18–35 years met the physical activity guidelines (20% vs. 50%).^37^

Moreover, a sedentary lifestyle due to the COVID-19 pandemic has led to an increase in obesity and overweightness.^39,40^ In comparison to pregnant women with a lower body mass index (BMI), pregnant women with a higher BMI were 1.4 times more likely to develop preeclampsia/eclampsia.^39^ This trend is in congruence with national studies.^28,39,41^ The risk of preeclampsia in Latin American women increased by over three times in overweight and obese women.^39^ Among Chinese women and in sub-Saharan Africa, studies have shown an association between maternal overweightness/obesity before pregnancy and an increased risk of HDP.^39,41^

Previous studies have also shown that COVID-19 affects physiopathological mechanisms and leads to increased reproductive complications and adverse pregnancy outcomes.^33,42^ COVID-19 may cause placental infection by binding to angiotensin-converting enzyme 2 receptors, leading to vasoconstriction and dysfunction of the renin–angiotensin system.^33^ In addition, a deleterious effect of COVID-19 occurs on placental vessels and can cause a leaky endothelium and thrombosis.^42^ Furthermore, COVID-19 in pregnancy led to infant morbidity, including respiratory distress and thrombocytopenia with abnormal liver function.^42^

Consistent with other studies,^1,16^ the present study concluded that African American women had the highest incidence of HDP. These racial and ethnic disparities may be attributed to differences in the quality of pre- and postnatal health care received.^17^ In addition, African American women have a greater prevalence of maternal morbidity and cardiovascular comorbidities (obesity, chronic hypertension, and preexisting diabetes), which are all major risk factors for HDP.^16^ Moreover, Minhas et al. showed that the morbidity ratio for African American women with HDP is disproportionately higher than White women with HDP (41.7 per 100,000 live births vs. 13.4 per 100,000 live births).^16^

Another prominent risk factor of HDP is a low preeclampsia, with the highest HDP mortality rate seen in areas of poverty.^29^ Many individuals with a low SES are a part of the Medicaid population. The mortality rate of HDP is inversely correlated with low total family incomes, with a significant aspect due to the role of economic support for both pregnant women and fetuses and disparities in prenatal health care.^29,43^ Many members of the Medicaid population have less access to quality health care and encounter numerous obstacles and challenges, such as health disparities due to social determinants of health, a lack of transportation to their medical appointments, and unaffordability of care due to a lack of insurance coverage.^44^ In addition, their quality of care pre- and postnatal periods may be decreased due to unawareness of critical information, sociodemographic factors (*e.g.*, level of education and marital status), and the rising cost of health care.^45^ Moreover, mothers with HDP and a low education level had a 49% higher risk of all-cause infant mortality compared with mothers with HDP alone.^28^ Thus, the Medicaid population is exceedingly vulnerable to HDP.

## Strengths and Limitations

The main strength of this present study is that a large cohort of pregnancies were obtained in the Louisiana Medicaid Program dataset to assess the trend in the incidence of HDP in the Louisiana Medicaid population. In addition, our dataset had a large sample size of African American and White women with available information on each type-specific HDP. However, some limitations to our findings are that they pertained to the Louisiana Medicaid population and may not translate well to the non-Medicaid population. Many risk factors are more prominent in the Medicaid population, which may contribute to observed discrepancies in the morbidity and mortality rate of diseases compared with the non-Medicaid population. Another limitation is the accuracy of ICD-10 coding to differentiate between gestational hypertension and chronic hypertension among women who are not evaluated early in pregnancy. However, the overall rates of HDP are likely to remain accurate, albeit with less accuracy attributed to each subtype.

## Conclusion

HDP remains a very prevalent and significant global health issue, especially in African American women and during the COVID-19 pandemic. Severe HDP substantially increases the risk of mortality in offspring and long-term issues in both the mother and infant. Therefore, an emphasis on preventative measures is crucial, such as increasing the quality of prenatal care, clinical evaluation, and counseling for better health outcomes, providing economic support (for low-income mothers), and raising more awareness for management of HDP. This is very pertinent to the Medicaid population due to the disparities and barriers that diminish the quality of health care they receive. Future studies can determine if there are any racial disparities present in the types of care women receive prenatally when they are at risk of HDP and how those disparities may affect both the short-term and long-term health of the infant.

## Data Sharing Statement

The datasets generated and analyzed during the current study are available from the corresponding author on reasonable request.

## Abbreviations Used

CMS: Centers for Medicaid Services
CVD: Cardiovascular disease
HDP: Hypertensive disorders of pregnancy
ICD-10: International Classification of Diseases, 10th revision
ISSHP: the International Society for the Study of Hypertension in Pregnancy
SES: socioeconomic status
